# T Cell Vaccination Inhibits Th1/Th17/Tfh Frequencies and Production of Autoantibodies in Collagen-Induced Arthritis

**DOI:** 10.1155/2013/967301

**Published:** 2013-12-02

**Authors:** Shan Li, Xiaoyin Niu, Yebin Xi, Shaohua Deng, Chengzhen Li, Qing Zhao, Guangjie Chen

**Affiliations:** ^1^Department of Immunology and Microbiology, Shanghai Jiao Tong University School of Medicine, Shanghai Institute of Immunology, Shanghai 200025, China; ^2^Breast Cancer Institute, Fudan University Shanghai Cancer Center, Shanghai 200032, China

## Abstract

The aim of this study is to determine whether the regulatory role of T cell vaccination (TCV) is through inhibition of Th1/Th17/Tfh and production of autoantibodies on collagen-induced arthritis (CIA). First, CIA mice were treated with TCV. After disease onset, the incidence and severity of change in joint histopathology were evaluated. Mice in the TCV-treated group showed less disease severity and less infiltration of inflammatory cells in the joint sections. TCV decreased the frequencies of Th1/Th17/Tfh cells and related cytokines. Reduction of IL-21 may be associated with both Tfh and Th17, which further influence B cell and T cell responses. In addition, inhibition of Th1/Th17/Tfh frequencies led to the reduced expression of T-bet, ROR**α**, ROR**γ**t, and Bcl6. Lastly, the proliferation of type-II-collagen-(CII-) specific T cells and the production of anti-CII antibodies were inhibited in the TCV-treated group. The results provide novel evidence that the therapeutic effects of TCV on CIA are associated with the inhibition of Th1/Th17/Tfh frequencies and autoantibodies production.

## 1. Introduction

Rheumatoid arthritis (RA) is an autoimmune disease of unknown etiology, characterized by the presence of inflammatory synovitis accompanied by the destruction of joint cartilage and bone [1]. Collagen-induced arthritis (CIA) represents an animal model of autoimmune polyarthritis with significant similarities to human rheumatoid arthritis that can be induced upon immunization with native type II collagen. Both CIA and RA are characterized by manifestations of cellular as well as humoral autoimmunity, which may act in concert to mediate disease progression.

T cell vaccination (TCV) has been reported to be effective in many autoimmune diseases, including experimental autoimmune encephalomyelitis and experimental arthritis [2–4]. TCV appears to induce regulatory immune responses through interactions of the host immune system with vaccine T cells, in both experimental animal models and humans [5]. It activates anti-idiotypic T cells by cytotoxic activity and antibody responses that react specifically with the T cell receptor (TCR) of vaccine T cells. It induces upregulation of Foxp3 expression and the inhibitory function of CD4+ CD25+ Tregs, which plays an important role in the regulation of autoreactive T cells and autoimmune diseases [6–9].

Recently, a body of evidence suggested that uncontrolled and persistent Th1, Th17 cells responses and their derived cytokines can contribute to autoimmune diseases, including RA [10]. Follicular helper T (Tfh) cells, a recently found subset of CD4+ T cells located in germinal centers (GCs), are characterized by persistently high expression of CXCR5 [11]. Tfh can also express other membrane molecules and secrete many cytokines, such as ICOS and IL-21, to participate in the development of B cells and thus regulate the secondary immune response to maintain immune balance [12, 13]. Upon exposure to a foreign antigen, Tfh cells help B cells generate antibody-producing plasma cells and long-lived memory B cells. B cell lymphoma 6 (Bcl6) is a transcription factor selectively expressed by Tfh cells and is regulated by IL-21 and IL-6. Deficiency of Bcl6 in T cells results in impaired Tfh cell development and GC reactions [13]. Studies have shown that unusually high amounts of Tfh cells are found in RA patients and experimental arthritis animals [14, 15].

High levels of various autoantibodies detected in RA patients trigger immune responses. This can activate many lymphocytes, such as macrophages, T cells, and B cells. In return, B cells may produce more antibodies to exacerbate the disease. Type II collagen (CII) is a critical autoantigen in RA. CII-specific antibodies are frequently found in RA patients [16–18]. Further, transfer studies have shown that autoantibodies are directly pathogenic and can provoke at least some of the manifestations of joint inflammation.

According to the above studies, we intended to reveal the regulatory role of TCV on CIA. In this study, we aimed to determine the effects of TCV on CIA. The data showed that T cell vaccine could delay onset of CIA, improve joint inflammation, and inhibit Th1/Th17/Tfh cells and related inflammatory cytokines. Additionally, the TCV could decrease proliferation of type-II-collagen-(CII-) specific T cells and production of autoantibodies. The findings described here provide novel evidence that the therapeutic effects of TCV on CIA were associated with the inhibition of Th1/Th17/Tfh frequencies and autoantibodies production. This study has important implications in the understanding of the role of TCV through cellular and humoral immunity in the inflammatory process of RA.

## 2. Materials and Methods

### 2.1. Ethnics Statements

The animal protocol used in this study was approved by the Institutional Review Board of Shanghai Jiao Tong University School of Medicine. All mice received humane care in compliance with the *Guide for the Care and Use of Laboratory Animals* published by the National Institutes of Health.

### 2.2. Animals

Male DBA/1 mice, 6–8 weeks of age and 20 ± 2 g, were purchased from Shanghai Slac Laboratory Animal Co. Ltd. (Shanghai, China) and housed in the animal care facility of Shanghai Jiao Tong University School of Medicine under pathogen-free conditions according to the Institutional Animal Care and Use Committee guidelines.

### 2.3. Induction and Assessment of CIA

Chicken type II collagen (CII, Sigma, St. Louis, MO, USA) was dissolved in 0.01 M acetic acid at 4°C overnight. The complete Freund's adjuvant (CFA) was prepared by mixing incomplete Freund's adjuvant (IFA, Sigma, St. Louis, MO, USA) and *Mycobacterium tuberculosis* (Strain H37RA, Difco, Detroit, MI, USA). The dissolved CII was then emulsified with an equal volume of CFA. At day 0, the mice were immunized subcutaneously (s.c.) at the base of the tail with 0.1 mL emulsion containing 150 *μ*g CII and 200 *μ*g Mycobacterium tuberculosis. At day 21, mice were boosted with emulsion of 50 *μ*g CII and IFA at the base of the tail. Mice were evaluated by two independent, blinded examiners every day using the following clinical score assessment system: Grade 0 = Normal; 1 = Mild, with definite redness and swelling of the ankle or wrist or apparent redness and swelling limited to individual digits, regardless of the number of affected digits; 2 = moderate redness and swelling of ankle or wrist; 3 = severe redness and swelling of the entire paw, including digits; and 4 = maximally inflamed limb with involvement of multiple joints.

### 2.4. Preparation of T Cell Vaccine

Spleen mononuclear cells (MNCs) were prepared from CIA mice by grinding through a nylon mesh. The resulting mononuclear cells were incubated at 5 × 10^5^ cells/well with irradiated antigen-presenting cells (APCs), in the presence of 50 IU/well recombinant mouse IL-2 and 20 *μ*g/mL CII in a 96-well plate. The RPMI 1640 contained 200 IU/mL penicillin, 200 IU/mL streptomycin, l M *β*2-mercaptoethanol, and 10% heat-inactivated fetal bovine serum. The culture medium was changed every 3 days and irradiated APCs were added every week. After 21 days of culture, cell lines were harvested and tested in proliferation assays. If CII antigen specificity of the cells was high, their phenotype was analyzed before irradiation.

### 2.5. Treatment Protocol

Mice were divided into three experimental groups, specifically Normal, Model (CIA mice), and TCV-treated (10 mice per group). T cell vaccine (1 × 10^7^ irradiated T cells each mouse) was administered subcutaneously two weeks before establishment of CIA. The CIA group of mice received PBS and served as a control.

### 2.6. Histologic Analysis

At the peak of CIA (about 35 days after first immunization), mice were sacrificed by cervical dislocation. The paws from 4 to 6 animals were randomly collected by two independent experimenters, fixed in 4% buffered-formaldehyde, decalcified in ethylenediaminetetraacetic acid (EDTA), embedded in paraffin, and cut into 4 *μ*m sections. The sections were then stained with hematoxylin and eosin (H&E). Histopathological changes were evaluated by optical microscope. Specifically, we assessed cell infiltration, cartilage destruction, and bone erosion.

### 2.7. Flow Cytometric Analysis

Three groups of mice were sacrificed at the peak of CIA. Drained lymph node (DLN) mononuclear cells (MNCs) were prepared and were labeled with FITC-conjugated anti-CD4, PE-conjugated anti-ICOS, Percp-cy5.5-conjugated anti-CXCR5 (BD Pharmingen, San Diego, CA, USA), or matched isotype controls for an additional 30 min. For intracellular IL-17 and IFN-*γ* staining, DLN MNCs were prepared and stimulated for 5 h with 50 ng/mL PMA (Sigma Aldrich, St. Louis, MO, USA), 750 ng/mL ionomycin (Calbiochem, La Jolla, CA, USA), and GolgiPlug at the recommended concentrations (BD Pharmingen, San Diego, CA, USA). Cells were stained with FITC-conjugated anti-CD4, fixed and permeabilized with Cytofix/Cytoperm solution (BD Pharmingen, San Diego, CA, USA), and then labeled with APC-conjugated anti-IFN-*γ* (eBioscience, San Diego, CA, USA), PE-conjugated anti-IL-17 (eBioscience, San Diego, CA, USA). Percentage of positive stained cells was analyzed using a FACS instrument (BD Biosciences, San Jose, CA, USA).

### 2.8. Cytokine Measurement

The levels of cytokines were determined by ELISA using IFN-*γ* (eBioscience, San Diego, CA, USA), IL-17 (Maibo Co., Ltd., Shanghai), and IL-21 (eBioscience, San Diego, CA, USA) kits. Three groups of mice were sacrificed at the peak of CIA. DLN MNCs were prepared. Briefly, 200 *μ*L aliquots of MNC (5 × 10^6^/mL) suspensions were added into 96-well round-bottom microtiter plates and were stimulated with CII (20 *μ*g/mL). After 48 h of incubation at 37°C in 5% CO_2_ and humified atmosphere, the supernatants were harvested. The cytokines of supernatants and sera were detected according the instructions of the ELISA kit.

### 2.9. CD4+ T Cell Isolation

At the peak of CIA, three groups of mice were sacrificed. CD4+ T cells were prepared from freshly isolated splenocytes using biotinylated CD4 antibody, then avidin binding Dynabeads, and subsequently Detachbeads (Dynal Biotech, New Hyde Park, NY, USA). The purity of CD4+ T cells was >99%, as determined by flow cytometry using specific antibodies.

### 2.10. RNA Isolation

Total RNA was isolated from CD4+ T cells (5 × 10^5^) using RNeasy Mini Kit (Qiagen). Genomic DNA was removed from total RNA prior to cDNA synthesis using RNase-free DNase Set (Qiagen). First-strand cDNA synthesis was performed for each RNA sample using Sensiscript RT Kit (Qiagen). Random hexamers were used to prime cDNA synthesis.

### 2.11. Real-Time RT-PCR Analysis of Gene Expression

Primer Express software (ABI) was used to design primers from published cDNA sequences. BLAST searches were conducted on the primer nucleotide sequences to ensure gene specificity. The primer sequences were as follows: *β*-actin, forward 5′-TTCAACACCCCAGCCATGT-3′ and reverse 5′-GTGGTACGACCAGAGGCATACA-3′, T-bet, forward 5′-forward 5′-GGTGTCTGGGAAGCTGAGAG-3′ and reverse 5′-TCTGGGGTCACATTGTTGGAA-3′, ROR*α*, forward 5′-TGCGAGCTCCAGCCGAGGTA-3′ and reverse 5′-GCCCTTGCAGCCTTCACACGTA-3′, ROR*γ*t, forward 5′-GGAGCTCTGCCAGAATGAGC-3′ and reverse 5′-CAAGGCTCGAAACAGCTCCAC-3′, and Bcl6, forward 5′-TGCACTTTCGTCACAAAAGC-3′ and reverse 5′-ATGCTTCATTCAGCAGGCTT-3′.

Relative quantification of gene expression was performed using the ABI Prism 7900 sequence detection system. SYBR Green master mix (ABI) was used for real-time RT-PCR to detect the abundance of PCR products among samples. Thermocycler conditions comprised of an initial holding at 50°C for 2 min, then 95°C for 10 min. This was followed by a 2-step PCR program consisting of 95°C for 15 s and 60°C for 60 s for 35 cycles. Data were collected and quantitatively analyzed on an ABI Prism 7900 HT sequence detection system (ABI). The *β*-actin gene was used as an endogenous control to normalize the differences in the amount of total RNA in each sample. All quantities were expressed as *n*-fold relative to a calibrator.

### 2.12. Proliferation Assay of CII-Specific T Cells

Three groups of mice were sacrificed at the peak of CIA. Spleen MNCs were prepared and incubated (5 × 10^5^ cells/well) in the absence and presence of heat-inactivated CII (20 *μ*g/mL) for 48 h to 54 h. [^3^H] Thymidine was added during the last 18 h of culture. The cpm value was then detected by liquid scintillation counter.

### 2.13. Immunoassay of Serum Anti-CII Antibody Level

Serum was collected from blood samples taken at the peak of CIA prior to any treatments. Anti-CII IgG, IgG1, and IgG2a levels were measured using ELISA. Wells of flat-bottom MaxiSorb microtiter plates were coated with 250 ng of CII in 50 *μ*L PBS at 4°C overnight. The plates were then blocked with 100 *μ*L PBS containing 1% bovine serum albumin and 0.05% Tween 20 at 37°C for 1 h. This was followed by 5 washes with PBS containing 0.05% Tween 20. Then, 50 *μ*L of serum diluted 1 : 5000 with PBS containing 1% bovine serum albumin and 0.05% Tween 20 was added. After 2 h incubation at 37°C, wells were washed 5 times with PBS containing 0.05% Tween 20 and then incubated at 37°C for 1 h with 50 *μ*L of a 1 : 1000 dilution (in PBS containing 1% bovine serum albumin and 0.05% Tween 20) of goat anti-mouse IgG, IgG1, and IgG2a coupled to HRP (eBioscience, USA). Following 5 more washes, 50 *μ*L TMB was added and stopped by 2 M H_2_SO_4_. The absorbance was then measured at 450 nm.

## 3. Statistical Analysis

A Student's *t*-test was used to analyze the differences between the groups. One-way ANOVA was initially performed to determine whether an overall statistically significant change existed before using the two-tailed paired or unpaired Student's *t* test. A value of *P* < 0.05 was considered statistically significant.

## 4. Results

### 4.1. T Cell Vaccination Decreased the Severity of CIA

**Figure 1:**
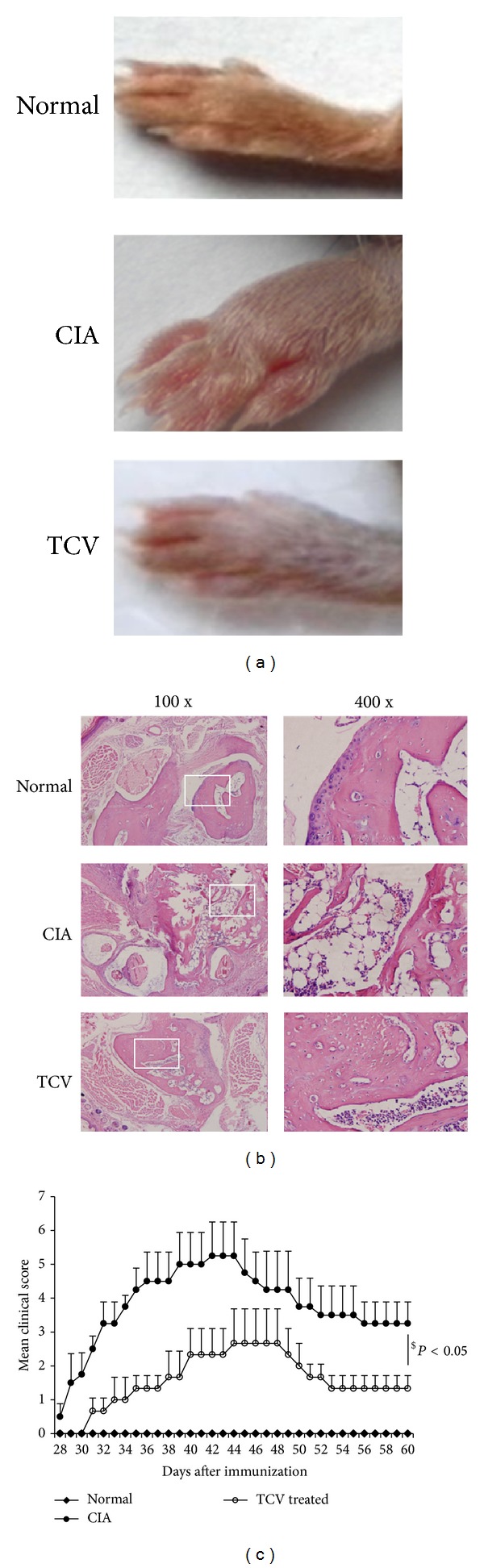
Clinical assessment of CIA and histopathological analysis of joints. The three experimental groups included normal, CIA, and TCV-treated groups. The TCV-treated group was immunized with 1 × 10^7^ irradiated T cells two weeks before the establishment of CIA. The CIA group was injected with PBS as a control. (a) Appearance of relevant paws. (b) Histopathological changes of joints. (HE, left ×100, right ×400). (c) Clinical scores were assessed. Data are represented as means ± SD (*n* = 10 mice/group). Data are representative of 5 separate experiments with similar results. ^$^ *P* < 0.05, TCV *versus* CIA group.

We evaluated the incidence of CIA in the mice after boost immunization. We assessed the activity of the mice, joint swelling, and the clinical score of the disease. Results showed that the incidence of the disease in the TCV-treated group had been reduced. The activity of TCV-treated mice was almost the same as that exhibited by mice in the normal group (Figure 1(a)). Histopathological sections showed serious bone destruction in the CIA control group, while it showed less inflammatory cell infiltration and lower bone destruction in the TCV-treated group (Figure 1(b)). The onset of CIA in control group mice started from day 28, while the onset of CIA in the TCV-treated group was delayed (Figure 1(c)). In addition, the clinical score of the latter group was significantly lower than that of the CIA control group, and the progress of the disease was also slower. At the peak of the disease (about day 35), clinical scores of mice in the TCV-treated group were lower than those of the CIA control group. In the latter stage of the disease, the clinical score of TCV-treated group was significantly lower than that of the CIA control group (*P* < 0.05; Figure 1(c)).

### 4.2. T Cell Vaccination Decreased the Frequencies of Th1/Th17/Tfh Cells and Related Cytokines

**Figure 2:**
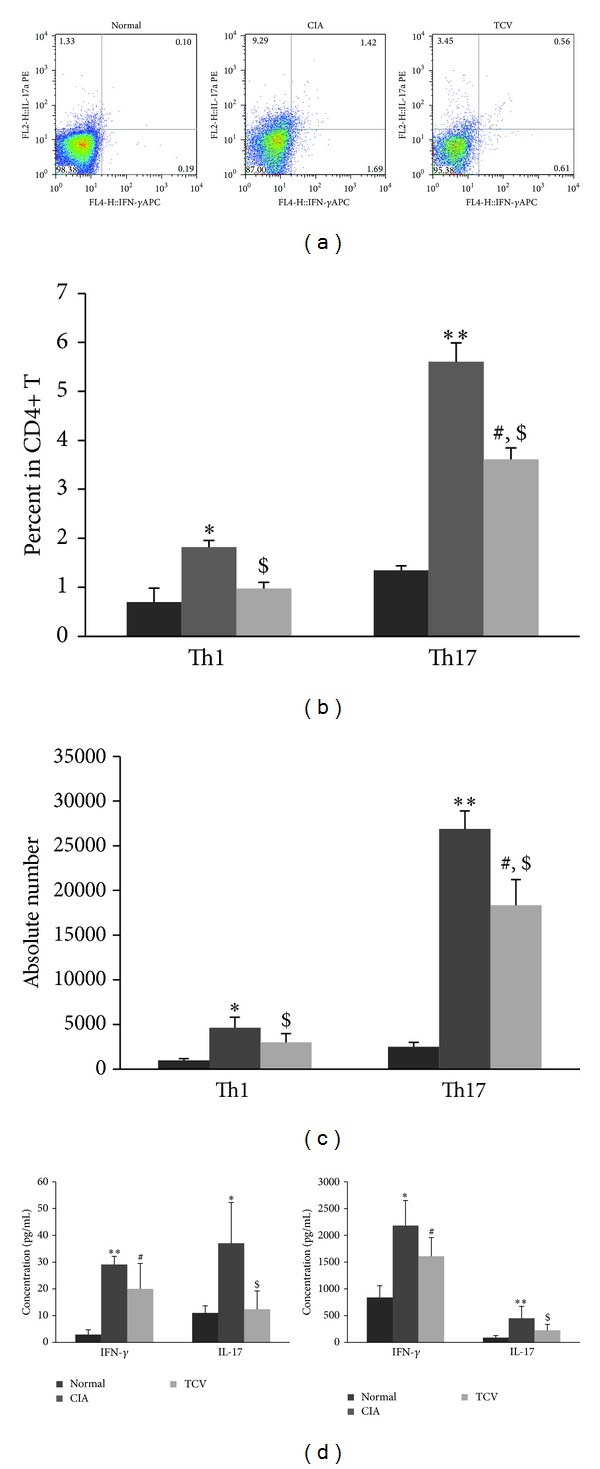
TCV influenced the frequency of Th1/Th17 cells and related cytokines in CIA mice. The three groups of mice were sacrificed at the peak of the disease. DLN MNCs were harvested. IFN-*γ* and IL-17 secreting cells were determined by intracellular staining and flow cytometry. Results showed the percentage of Th1/Th17 cells in DLN CD4+ cells (a)-(b). (c) Results showed the absolute number of Th1/Th17 cells in DLN. (d) Heart blood was collected and the serum was isolated by centrifuge. DLN MNCs were prepared and incubated (5 × 10^5^ cells/well) in the absence and presence of heat-inactivated CII (20 *μ*g/mL) for 48 h and the supernatants were collected. The levels of IFN-*γ* and IL-17 were determined using ELISA. Concentration of IFN-*γ* and IL-17 in sera (left) and supernatants of cell culture (right). Data are represented as means ± SD (*n* = 10 mice/group). Data are representative of 5 separate experiments with similar results. *P < 0.05, **P < 0.01, CIA *versus* normal group. ^#^ *P* < 0.05, TCV versus normal group. ^$^ *P* < 0.05, TCV versus CIA group.

As we know, the activities of inflammatory cells and related cytokines play important roles in the whole periods of arthritis, such as the infiltration of Th1 and Th17 cells in the joints. In Figures 2(a)-2(b), the percentages of Th1 and Th17 cells in CD4+ T cells in TCV-treated group are much lower than those in CIA control group. We also calculate the absolute number of Th1 and Th17 cells in DLN. The numbers of Th1 and Th17 cells in TCV-treated group are much lower than those in CIA control group too (Figure 2(c)). We next investigate cytokines secreted by those two Th subsets, IFN-*γ* and IL-17. Data shows that both of them are suppressed in whatever sera of mice or supernatants of cell culture (Figure 2(d)). In a conclusion, T cell vaccine prevents the progression of CIA by strong reduction of inflammatory response and downregulating the production of several inflammation mediators in the joint and DLN.

**Figure 3:**
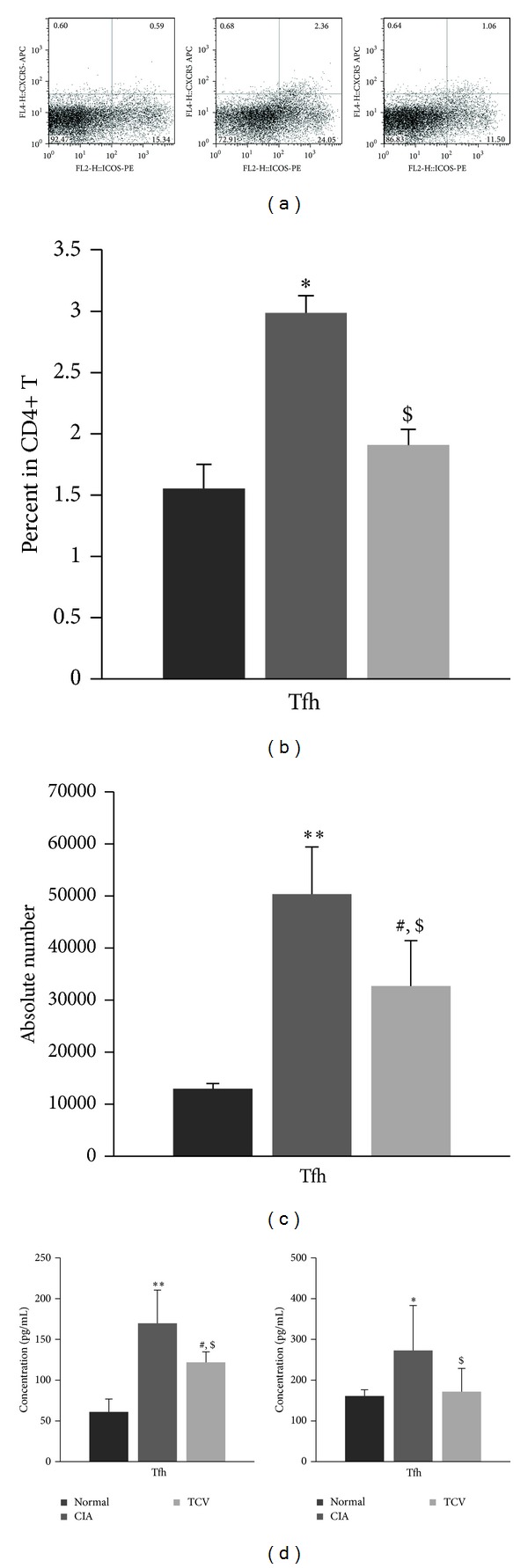
TCV influenced the frequency of Tfh cells and IL-21 in CIA mice. The three groups of mice were sacrificed at the peak of the disease. DLN MNCs were harvested, stained with antibodies for CD4, CXCR5, and ICOS, and analyzed by flow cytometry. (a)-(b) Flow cytometry results showed the percentage of Tfh cells in DLN CD4+ cells. (c) Results showed the absolute number of Th1/Th17 cells in DLN. (d) Heart blood was collected and the serum was isolated by centrifuge. Spleen MNCs were prepared and incubated (5 × 10^5^ cells/well) in the absence and presence of heat-inactivated CII (20 *μ*g/mL) for 48 h and the supernatants were collected. The levels of IL-21 were determined using ELISA. Concentration of IL-21 in sera (left) and supernatants of cell culture (right). Data are represented as means ± SD (*n* = 10 mice/group). Data are representative of 5 separate experiments with similar results. *P < 0.05, **P < 0.01, CIA *versus* normal group. ^#^ *P* < 0.05, TCV versus normal group. ^$^ *P* < 0.05, TCV versus CIA group.

Tfh cells are the main cells that help B cells produce antibodies. We detected them by FACs and found that the frequency of Tfh cells among CD4+ T cells of the CIA group was much higher than that of the normal group, while it was reduced in the TCV-treated group, as well as the absolute number of Tfh cells (Figures 3(a)–3(c)).

Tfh cells can secrete large amounts of cytokines to help develop B cells and themselves. Thus, we detected the level of IL-21, the main cytokine produced by Tfh, in the sera of the mice and supernatant of the cell cultures. The results showed that TCV treatment can significantly inhibit the level of IL-21 (Figure 3(d)).

### 4.3. T Cell Vaccination Led to the Reduced Expressions of T-bet, ROR*α*, ROR*γ*t, and Bcl6

**Figure 4:**
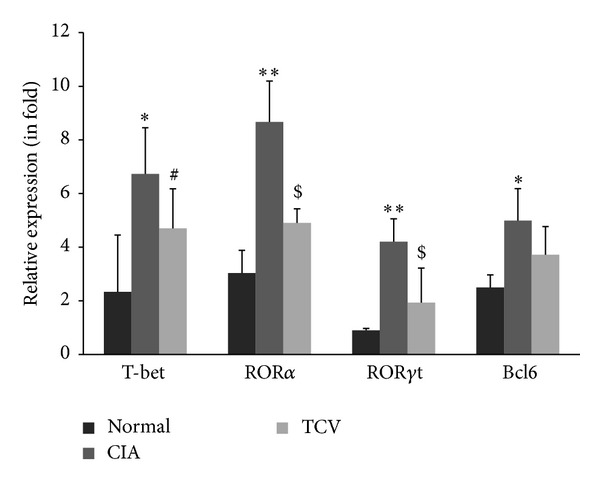
TCV influenced the expressions of Th1/Th17/Tfh related transcriptional factors in CIA mice. Purified CD4+ T cells from splenocytes were prepared and analyzed for the mRNA expressions of T-bet, ROR*α*, ROR*γ*t and Bcl6 by real-time RT-PCR. Data are presented as means ± SE. Data are represented as means ± SD (*n* = 10 mice/group). Data are representative of 5 separate experiments with similar results. *P < 0.05, **P < 0.01, CIA *versus* normal group. ^#^ *P* < 0.05, TCV versus normal group. ^$^ *P* < 0.05, TCV versus CIA group.

We further addressed the underlying mechanisms by examining the mRNA levels of T-bet, ROR*α*, ROR*γ*t, and Bcl6 in three groups. It was evident that T cell vaccination markedly suppressed expressions of T-bet, ROR*α*, ROR*γ*t, and Bcl6 (Figure 4).

### 4.4. T Cell Vaccination Inhibited the Proliferation of CII-Specific T Cells and Production of Anti-CII Antibodies

**Figure 5:**
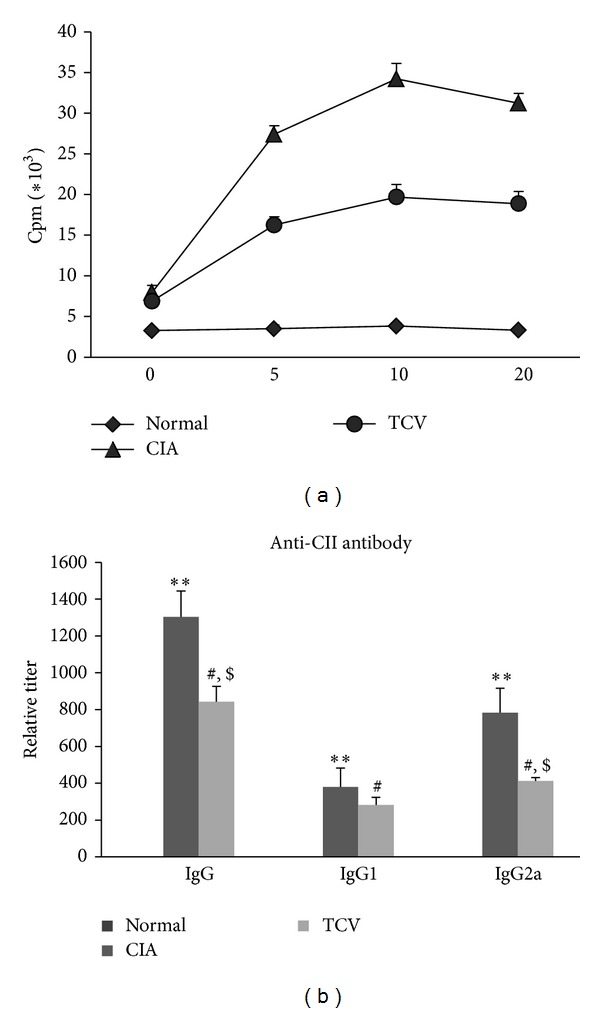
TCV influenced the proliferation of CII-specific cells and levels of CII antibodies in CIA mice. The three groups of mice were sacrificed at the peak of the disease. Spleen MNCs were prepared and incubated (5 × 10^5^ cells/well) in the absence and presence of heat-inactivated CII (20 *μ*g/mL) for 48 h to 54 h. [^3^H]Thymidine was added during the last 18 h of culture. (a) The cpm values were obtained by liquid scintillation counter in the proliferation assay. (b) Heart blood was collected at the peak of disease and the serum was isolated by centrifuge. The levels of IgG, IgG1, and IgG2a were detected by indirect ELISA. Data are represented as means ± SD (*n* = 10 mice/group). Data were representative of 3 separate experiments with similar results. *P < 0.05, **P < 0.01, CIA *versus* normal group. ^#^ *P* < 0.05, TCV versus normal group. ^$^ *P* < 0.05, TCV versus CIA group.

In order to determine whether impaired T cell function in TCV-treated mice led to CIA inhibition, we used an H3-TdR proliferation test to detect the response of spleen lymphocytes to collagen. The results showed that T cells from mice receiving TCV responded to CII in a much lesser extent (Figure 5(a)).

High levels of circulating antibodies directed against CII invariably accompany the development of CIA and seem to be required for disease development. Thus, the production of antibodies against CII is a major factor in determining susceptibility to CIA. Because the development of antigen-specific antibodies requires Tfh help, one mechanism of CIA inhibition by TCV could be due to the failure to produce antibodies against CII, particularly autoreactive IgG2a antibodies that have been implicated in the pathogenesis of CIA. We measured the serum levels of total IgG or isotype-specific IgG2a and IgG1 anti-CII antibodies at the peak of the disease. CIA resulted in high levels of CII-specific IgG antibodies, characterized by a high level of IgG2a. In contrast, treatment of CIA mice with TCV significantly reduced CII-specific IgG and IgG2a levels (Figure 5(b)).

## 5. Discussion

Rheumatoid arthritis is a systemic inflammatory disease, presumably of autoimmune origin. Due to its pathological, immunological, and clinical similarities to human RA, CIA is a commonly used model for studying RA and testing potential therapeutic agents [19, 20]. In this study, we demonstrated that T cell vaccination is effective on CIA, as our data showed the disease state of in the TCV-treated group significantly improved.

We showed that TCV can delay the onset of disease, reduce the clinical scores of arthritis, and decrease the incidence of CIA. H&E staining showed that TCV treatment can decrease infiltration of inflammatory cells and protect the bone and cartilage system in joints from damage. Additionally, the activity of mice in the TCV group was observed to be nearly the same as that in the normal group. All these data showed that TCV has great effect on CIA.

TCV was first discovered for the treatment of experimental autoimmune encephalomyelitis (EAE). Mice immunized with autoimmune T cells can develop the same symptoms as EAE induced by myelin basic protein (MBP) antigen. Therefore, vaccination with these irradiated T cells can inhibit pathological response by inducing the regulatory network of the immune system [2]. It is known that the immune system can regulate itself by recognizing T cell receptors expressed on the surface of a T cell vaccine. During the process, TCV functions by inducing anti-idiotype and antiergotype responses. These responses are mediated by CD8+ T and CD4+ T cells, respectively [6, 21]. In our study, we demonstrated that TCV treatment can upregulate regulatory CD4+ T cells (data not shown) and the secretion of IL-10, which is the main component in the suppression of autoimmune cells.

We also analyzed the different frequencies of T cell subsets, including Th1, Th1 7cells in CIA after TCV treatment for the first time. As previously described, the Th1 and Th17 subsets have a crucial role in RA pathology [10]. As it is known, Th17 cells, which can be differentiated and proliferated from CD4+ T cells under the stimulation of IL-1 and IL-21, produce IL-17, IL-1, IL-6, IL-21, and TNF-*α*. These proinflammatory cytokines play multifaceted roles in autoimmune inflammatory processes [22]. In this study, we showed that TCV treatment may inhibit the development of RA and significantly decrease the percentage of Th1, Th17 cells and their related cytokines IFN-*γ*, IL-17. The suppression of Th1 and Th17 cells may be associated with antiidiotype and antiergotype regulatory responses.

Some studies find that TCV can also decrease the levels of autoantibodies in different autoimmune diseases [3, 23]. High levels of circulating antibodies directed against autoantigens invariably accompany the development of autoimmune diseases and seem to be required for disease development. These findings suggest that the effect of TCV on autoimmune diseases not only influences the cellular immune system but also regulates the immune system by humoral immunity. Recently, Tfh cells were found to help B cells generate antibody-producing plasma cells and long-lived memory B cells. Overexpressed Tfh cells and related cytokines most likely contribute to the pathogenesis of certain autoimmune diseases. According to these findings, we wondered whether TCV could influence Tfh cell, as well as cause decreased level of CII-specific antibody in CIA. So, we then detected the percentages of Tfh in CD4+ T cells. Tfh cells are typically identified by their expression of cell surface markers, such as PD-1 and/or ICOS, in conjunction with CXCR5, which has been used as a proxy for location within the follicle. The results showed that the percentage of CXCR5+ ICOS+ CD4+ Tfh cells in the CIA group is much higher than that in the normal group, while TCV treatment can reduce that percentage significantly.

Tfh cells can secrete large amounts of cytokines, to help in the development of B cells and themselves. The main related cytokine is IL-21, which functions by autocrine secretion to stimulate a T-B reaction [24]. Within the CD4+ T cell subset, IL-21 is expressed at the highest levels by T follicular helper (Tfh) cells and Th17 cells [25–28]. IL-21 promotes antibody production, plasma cell differentiation, and switching to IgG1 in the context of thymus-dependent (TD) responses. Thus, we detected the level of IL-21 in the sera of the mice and supernatant of the cell cultures. The results showed that TCV can significantly inhibit the level of IL-21.

T-bet, ROR*α*, and ROR*γ*t are important players during Th1 and Th17 differentiation, respectively. Our results showed that the mRNA expressions of T-bet, ROR*α*, and ROR*γ*t were significantly decreased after TCV treatment in CIA mice. In naive T cells, IL-21 leads to upregulation of Bcl6, the transcriptional regulator of Tfh cells [29]. Bcl6 expression is induced by IL-21 and can lead to the expression of CXCR5, a homing molecule for the germinal center. Our results showed that the mRNA expression of Bcl6 decreased in CD4+ T cells of the TCV-treated group.

At last, we detected the response of spleen lymphocytes to collagen by proliferation test. The results showed that T cells from mice receiving TCV responded to CII in a much lesser extent. These data indicate that TCV during CIA development at least partially inhibits T cell clonal expansion in response to CII challenge. We further measured the serum levels of total IgG and isotype-specific IgG2a and IgG1 anti-CII antibodies at the peak of the disease. CIA resulted in high levels of CII-specific IgG antibodies, characterized by a high IgG2a level. In contrast, treatment of CIA mice with TCV significantly reduced CII-specific IgG levels. Interestingly, the reduction of IgG2a was more significant than the reduction of total IgG. This implies that TCV may influence antibody conversion. We speculate that the role might be performed by Tfh cells. Because the development of antigen-specific antibodies requires Tfh cell help [30], one mechanism of CIA inhibition by TCV could be due to the failure to produce antibodies against CII, particularly autoreactive IgG2a antibodies that have been implicated in the pathogenesis of CIA. Dampening IL-21 signaling may be useful to diminish the production of high affinity autoantibodies.

In summary, the therapeutic effects of TCV on CIA could be due to different mechanisms that are not mutually exclusive. Our results imply that the regulatory role of TCV during CIA involved the decreasing of Th1/Th17/Tfh frequencies and production of autoantibodies. This study has important implications in understanding the role of TCV through cellular and humoral immunity in the inflammatory process of RA.

## Supplementary Material

Supplemental Figure 1: Clinical assessment of CIA after adoptive transfer of CD4, CD4*+*CD25- and CD4*+*CD25*+* T cells.
