# Supplementary material for: T Cell Vaccination Inhibits Th1/Th17/Tfh Frequencies and Production of Autoantibodies in Collagen-Induced Arthritis
Source: Clin Dev Immunol. 2013 Dec 2;2013:967301. doi: 10.1155/2013/967301 (PMC3865644; doi:10.1155/2013/967301)
Supplement: Supplementary file 1 — Supplemental Figure 1: Clinical assessment of CIA after adoptive transfer of CD4, CD4+CD25- and CD4+CD25+ T cells. [file 967301.f1.pdf]

Supplemental Figure 1. Clinical assessment of CIA after adoptive transfer of CD4, CD4+CD25- and CD4+CD25+ T cells. The two experimental groups were transferred different T cell subsets from CIA and TCV-treated mice. Mice of each group were adoptive transferred with CD4+, CD4+CD25- and CD4+CD25+ T cells, respectively. After 14 days, clinical scores were assessed. Data are represented as means ± SD (n=5 mice/group). Data are representative of 3 separate experiments with similar results. *P<0.05, CD4+CD25- *vs.* CD4+T cells adoptive transfer. $^{\#}$P<0.05, CD4+CD25+ *vs.* CD4+T cells adoptive transfer. $^{\$}$P<0.05, CD4+CD25- *vs.* CD4+CD25-T cells adoptive transfer.

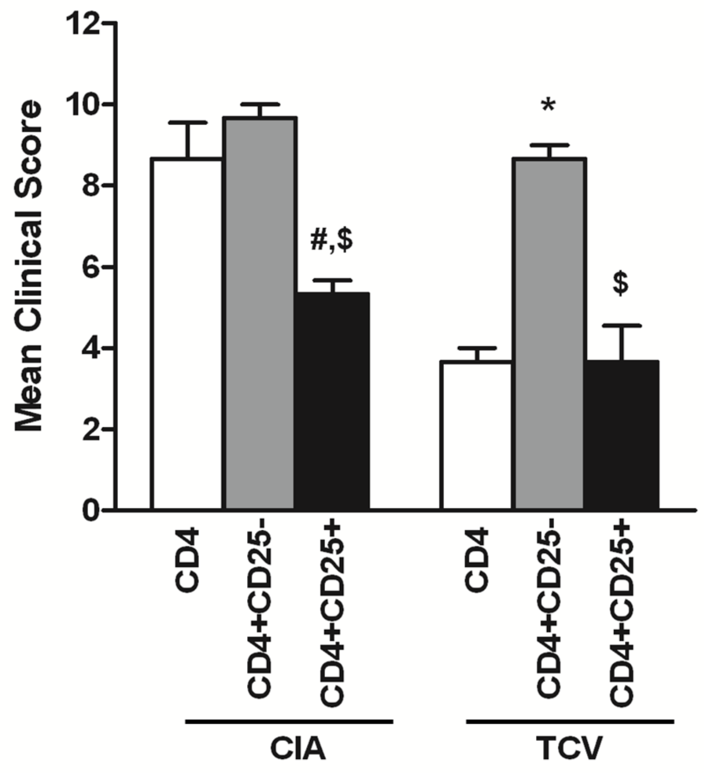

Supplemental Fig 1.
